# The New Microbiology: From Microbiomes to CRISPR

**DOI:** 10.3201/eid2806.212085

**Published:** 2022-06

**Authors:** Tomi Obe, Nikki W. Shariat

**Affiliations:** University of Georgia, Athens, Georgia, USA

**Keywords:** microbiology, microbiomes, infections, CRISPR

Infections, diseases, epidemics, and pandemics are some of the words that come to mind when one hears the word “microbes.” Although microbes, including bacteria and viruses, are responsible for many infections, they are beneficial for maintaining good health and environmental stability. The quest to understand the interaction between microorganisms and the environment led microbiologists to major discoveries of the 20th century, including DNA, microbiomes, and clustered regularly interspaced short palindromic repeats (CRISPR).

The New Microbiology: From Microbiomes to CRISPR by Pascale Cossart, a microbiologist at the Institut Pasteur (Paris, France), presents an engaging background of revolutionary discoveries and advances in microbiology (Figure). The author uses her extensive knowledge of bacteriology to offer a succinct and compelling narrative on the evolution of microbiology over the centuries.

**Figure:**
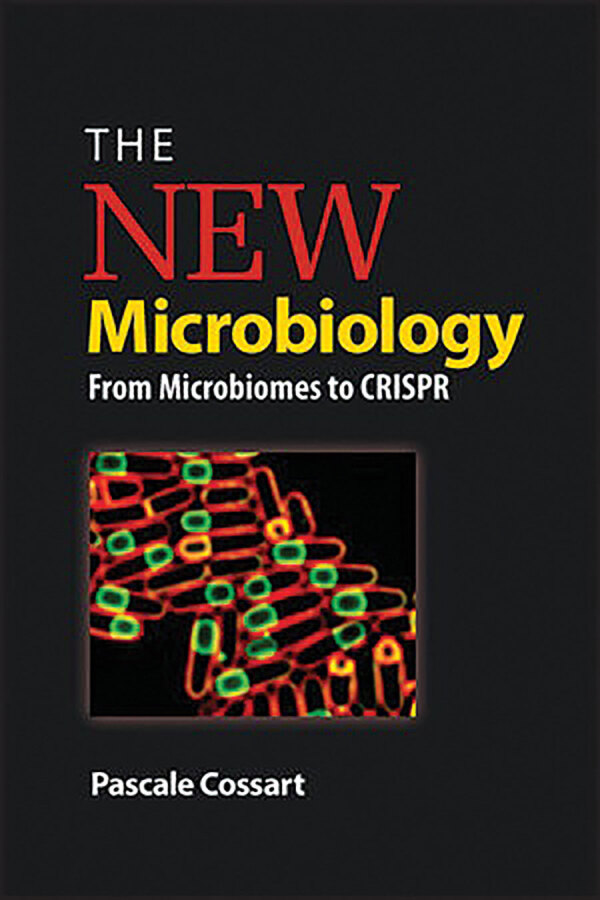
The New Microbiology: From Microbiomes to CRISPR

Each of the 4 parts of this book details certain advances in microbiology. In the first part (New Concepts in Microbiology), Cossart presents a broad and informative discussion that covers bacteria–host interaction, contributions to the ecosystem, and survival mechanisms in the environment. The CRISPR and CRISPR/Cas9 systems used for genome modification in prokaryotes and eukaryotes is a notable discovery detailed by the author. In the second section (Sociomicrobiology: the Social Lives of Bacteria), the author describes how bacteria can exist as either single species or multispecies in communities called biofilms that are formed through adherence to surfaces. Her discussion includes how these biofilms form, how bacteria can communicate through specific signals, and different methods by which some bacteria thrive. For example, she notes that “*Bdellovibrio* can invade other bacteria and multiply, causing the host bacteria to explode,” but also points out that some other microbial communities in specific environments, known as microbiotas, “produce innumerable compounds that benefit its inhabitants.”

Cossart explores the discovery and pathogenicity of different disease-causing bacteria in the book’s third section (The Biology of Infections). She details historical plagues such as the bubonic plague, caused by *Yersinia pestis* bacteria, and other bacterial diseases, including pertussis and diphtheria. She describes the evolution of other bacteria that are either foodborne, sexually transmitted, or pathogenic to insects and plants. In the book’s conclusion (Bacteria as Tools), she elucidates ways bacteria are being used as tools for research in different contexts. Cossart thoroughly highlights the most revolutionary discoveries in microbiology, including PCR technology that detects and amplifies DNA fragments from small sample and the CRISPR/Cas9 system, a genome modification and editing technology that “could generate bacteria capable of synthesizing medicines or their precursors on a massive scale.”

This book provides a straightforward, in-depth assessment of microbiology concepts. Readers will be fascinated with the detailed description of microbial evolution and the connection between old and new concepts. Although considerable advances have occurred in microbiology, such as the use of novel molecular tools to clarify mechanisms underlying bacteria–host interaction, the author offers compelling arguments that show microbiology will continue to evolve and that new discoveries are on the horizon. Cossart provides convincing evidence that some of these discoveries will enable us to evaluate the role of different microbiotas in host defense, whereas other discoveries will uncover ways to protect the environment from imminent dangers, including those associated with climate change.

